# Exploring Nanoplastics Bioaccumulation in Freshwater Organisms: A Study Using Gold-Doped Polymeric Nanoparticles

**DOI:** 10.3390/nano15020116

**Published:** 2025-01-15

**Authors:** Gabriella F. Schirinzi, Guillaume Bucher, Marisa Sárria Pereira de Passos, Vanessa Modesto, Miguel-Ángel Serra, Douglas Gilliland, Nicoletta Riccardi, Jessica Ponti

**Affiliations:** 1 European Commission, Joint Research Centre (JRC), 21027 Ispra, Italy; gabriella.schirinzi@ec.europa.eu (G.F.S.); guillaume.bucher@ec.europa.eu (G.B.); m.sarria@biotec.rwth-aachen.de (M.S.P.d.P.); miguel.serra-beltran@ec.europa.eu (M.-Á.S.); douglas.gilliland@ec.europa.eu (D.G.); 2 Water Research Institute (IRSA), National Research Council (CNR), 28922 Pallanza, Italy; vane.modesto@gmail.com (V.M.); nicoletta.riccardi@irsa.cnr.it (N.R.)

**Keywords:** nanoplastics, gold nanoparticles, bioaccumulation, freshwater organisms, ICP-MS, electron microscopy

## Abstract

The evaluation of nanoplastics bioaccumulation in living organisms is still considered an emerging challenge, especially as global plastic production continues to grow, posing a significant threat to humans, animals, and the environment. The goal of this work is to advance the development of standardized methods for reliable biomonitoring in the future. It is crucial to employ sensitive techniques that can detect and measure nanoplastics effectively, while ensuring minimal impact on the environment. To understand nanoplastics retention by freshwater organisms, phyto- and zooplankton, and mussels were exposed to gold-doped polymeric nanoparticles synthesized in our laboratory. The results demonstrated that measuring gold content using inductively coupled plasma mass spectrometry (ICP-MS), along with confirmation of its presence through electron microscopy in selected exposed samples provides insight into the accumulation and release of nanoplastics by organisms playing a relevant ecological role at the early levels of aquatic food webs.

## 1. Introduction

Freshwater ecosystems are vital components of the global environment, supporting diverse biological communities and providing essential services such as drinking water, irrigation, and habitat for countless species. Among these, freshwater model organisms—e.g., *Daphnia magna*, mollusks, zebrafish (*Danio rerio*), and various species of aquatic worms—play a critical role in understanding ecological dynamics and environmental impacts, serving as bioindicators of pollution and other ecological stressors [1,2]. These organisms are widely used for aquatic risk assessment and in environmental toxicology due to their sensitivity to chemical pollutants including microplastics, ease of culture, and their key ecological roles in aquatic food webs [3].

Recently, there has been growing concern about the accumulation of sub-micrometric particles, including nanoplastics, in freshwater ecosystems [4,5]. Nanoplastics are tiny plastic particles, typically less than 1 µm in size, that arise from the degradation of larger plastic debris (micro- or macroplastics) or are intentionally manufactured for use in various industries. Due to their small size and distinct physical and chemical properties, they exhibit a strong tendency to interact with natural organic matter and biological tissues, which could result in bioaccumulation and harmful effects on aquatic organisms [6,7]. Freshwater organisms, due to their close association with sediment or their ability to filter large volumes of water over extended periods, may be particularly susceptible to the uptake of nanoparticles. This makes them ideal candidates for studying nanoplastics bioaccumulation and assessing potential ecological risks.

The bioaccumulation of plastics in aquatic organisms is typically studied through controlled experimental exposure combined with various analytical techniques to detect and track the uptake, distribution, and concentration of nanoplastics in tissues or the surrounding environment [8,9]. Common methodologies include electron microscopy, Raman spectroscopy, and Pyr-GC/MS for the identification of nanoplastics in complex matrices [10]. However, these techniques are often time-consuming, can be affected by the presence of the biological residues and may not be suitable for nanoscale particles under realistic exposure conditions due to limitations in detection methods [11]. Alternative methods, such as imaging with fluorescently labeled nanoplastics, are commonly used in toxicology studies to track the uptake and distribution of particles within organisms. However, these methods have often been limited to qualitative identification, and lack the ability to provide precise quantitative measurements [12,13,14].

More recently, a novel approach using metal-doped nanoplastics has emerged. These particles can be effectively tracked using sensitive trace metal analysis methods, such as inductively coupled plasma mass spectrometry (ICP-MS) and single particle ICP-MS (spICP-MS); and have been successfully applied in multiple studies to evaluate the biological fate and uptake of nanoplastics [15,16,17,18].

In this study, the uptake and release by different freshwater organisms collected from Lake Maggiore (Varese, Italy), such as microalgae, zooplankton (*Daphnia longispina-galeata* species complex and *Eudiaptomus padanus;* Burckhardt, 1900), and mussels (*Unio elongatulus;* Pfeiffer, 1825), were evaluated when exposed to a test nominal concentration of 10^8^ particles mL^−1^ of three gold-doped polymeric nanoparticles (Au-PE, Au-PP, and Au-PVC).

These freshwater organisms were selected because they represent the earliest trophic levels in lentic waters and constitute the largest fraction of the biomass in these ecosystems. In particular, environmental microalgae, as primary producers, form the foundation of aquatic food webs by generating organic matter and oxygen. Zooplankton is the food link between primary producers and higher trophic levels (mainly fish) in the pelagic domain. Mussels, such as *Unio elongatulus,* play a crucial role for bentho-pelagic coupling and are widely recognized as efficient bioaccumulators [19,20], being therefore highly applied as environmental models to detect nanoparticles’ effects [21,22].

Understanding how these freshwater organisms respond to nanoparticle exposure can provide valuable information on the bioaccumulation, trophic transfer, and potential risks associated with plastic contamination in aquatic environments.

## 2. Materials and Methods

### 2.1. Chemicals

Ultrapure water (UPW, 18.2 MΩ.cm at 25 °C) was obtained using a Milli-Q water station from Merck Millipore (Molsheim, France).

Gold (Au), Indium (In), and Yttrium (Y) standard solutions for ICP-MS analysis at 1000 mg L^−1^, as well as hydrogen peroxide (30% H_2_O_2_ for trace analysis), concentrated HNO_3_ (69% TraceSELECT) and concentrated HCl (36% Suprapur) were supplied by Sigma Aldrich (Merk Life Science S.r.l., Milano, Italy).

The Ultra Uniform Gold Nanospheres (50 nm) were purchased from nanoComposix (San Diego, CA, USA).

### 2.2. Characterisation of Gold-Doped Polymeric Nanoparticles for Testing

Three types of gold-doped nanoplastics, namely, Au-PVC, Au-PE, and Au-PP, were employed to assess bioaccumulation patterns. These nanoplastics were synthesized in-house at the JRC according to the protocol previously described by Cassano et al. 2021 and 2023 [23,24], and further characterized to verify their long-term stability. 

The plastic nanoparticles were synthesized incorporating ultrasmall gold nanoparticles (Au-NPs, diameter ≈ 3 nm). The size distribution of the freshly synthetized gold-doped nanoplastics ranged from 50 to 350 nm for Au-PE and Au-PVC, and ranged from 100 to 400 nm for Au-PP.

Each type of gold-doped polymeric nanoparticle was tested for stability in culture medium (0.22 µm filtered freshwater from Lake Maggiore, Varese, Italy) for an incubation time longer than the exposure time selected. Stability tests were performed to check for possible release of “free” ultrasmall gold nanoparticles, particle size, and aggregation state in the exposure medium, for 6 days of incubation.

#### 2.2.1. Assessment of “Free” Ultrasmall AuNPs by Inductively Coupled Plasma Mass Spectrometry (ICP-MS)

To collect potentially ’free’ Au nanoparticles present in stock suspensions, gold-doped nanoplastics (Au-PVC, Au-PE and Au-PP) were filtered using 20 nm pore size Whatman Anotop syringe filters (Cytiva Italy SRL, Buccinasco, Italy), after ensuring that this type of filter does not retain ultrasmall Au-NPs used for doping these nanoplastics. Unfiltered suspensions and filtrates were digested with concentrated aqua regia (HNO_3_:HCl 1:3 *v*/*v*) for 60 min at 80 °C.

Once cooled down to room temperature, internal standard stock solution (Y and In at 0.1 mg L^−1^) was added and the digests were appropriately diluted with UPW to reach a final concentration of 8% aqua regia and 1 µg L^−1^ Y and In, prior to ICP-MS analysis.

Total Au determination was performed with a 7700x ICP-MS (Agilent Technologies, Santa Clara, CA, USA) equipped with a Micromist nebulizer, a Scott double-pass spray chamber set at 2 °C, a quartz torch with 2.5 mm internal diameter injector, platinum sampling and skimmer cones, and MassHunter 5.2 software version D.01.02 Build 708.1 (instrument configuration details available in SI Table S1).

Instrument performance (sensitivity, oxide, and doubly charged ion ratios) was checked daily after optimization of the measurement conditions using a standard built-in software procedure, and a multi-elemental tuning solution.

All the digests were analyzed against a ^197^Au calibration curve made up of seven points (0–25–50–100–500–1000–5000 ng L^−1^), with ^115^In at 1 µg L^−1^ as an internal standard in 8% aqua regia. The Limit of Quantification (LoQ) was set as the first positive calibration point, i.e., 25 ng L^−1^ in the injected solution.

For validation purposes and quality control (QC), the following criteria were systematically checked: (i) calibration curve linearity (R^2^ ≥ 0.995) and accuracy on concentrations (70% to 130% at LoQ and 90% to 110% for concentrations above LoQ); (ii) internal standard recovery for standard solutions and samples (within 90–110%); (iii) Au concentration in blanks injected between samples < 50 ng L^−1^ (2*LoQ); (iv) QCs (Au standard solution) recovery within 70–130% at 50 ng L^−1^ and within 90–110% at 500 ng L^−1^.

#### 2.2.2. Stability Test in the Exposure Medium

To measure the stability of gold-doped nanoplastics in exposure medium, in terms of size-distribution and aggregation state, each suspension was diluted and incubated for 6 days at room temperature in filtered freshwater from Lake Maggiore (Varese, Italy), to reach the exposure nominal concentration of 10^8^ particles mL^−1^_._ The suspensions were then analyzed by DLS as-is, and by spICP-MS following appropriate dilution in UPW.

spICP-MS analysis was performed with a Nexion 300D ICP-MS (Perkin Elmer, Waltham, MA, USA) equipped with an SC Fast peristaltic pump, a Meinhard concentric nebulizer, a glass cyclonic spray chamber and a standard quartz torch (2.5 mm id) (instrument configuration details available in SI Table S2).

The instrument was operated in standard mode (No Gas) with Syngistix and Nano Application version 2.5. Instrument performance (sensitivity, oxide, and doubly charged ion ratios) was checked daily after optimization of the measurement conditions using a standard built-in software procedure and a multi-elemental tuning solution.

Measurements were performed with a dwell time of 100 µs, monitoring ^197^Au and with an acquisition time of 60 s. Once the appropriate Au ionic (0–1–3–10 µg L^−1^ in UPW) and particulate (50 nm Ultra Uniform Gold Nanospheres in UPW) calibrations were performed, the transport efficiency was determined using the “particle size” method [25,26]. The Au-based Equivalent Spherical Diameter (ESD) of the individual gold-doped nanoplastics was calculated directly by the Nano application of the Syngistix software using a mass fraction of 100% and a density of 19.3 g cm^−3^ for gold. DLS measurements of the hydrodynamic diameter (Z-average) of gold-doped nanoplastics in filtered freshwater (exposure medium) were performed with a Zetasizer Nano ZS (Malvern Panalytical Ltd, Malvern, United Kingdom) at 23 °C in low-volume disposable cuvettes with six replicate measurements of 10 s each, averaged per recording.

### 2.3. Exposure of Freshwater Model Organisms

Environmental microalgae, zooplankton (*Daphnia longispina-galeata* group and *Eudiaptomus padanus*), and mussels (*Unio elongatulus*) were selected as freshwater model organisms. These were collected from Lake Maggiore (Varese, Italy) to test their interaction with nanoplastics under environmental-like exposure conditions (15 °C, dark, agitation, and oxygen; Innova42 incubator, Eppendorf, Italy).

The freshwater organisms were adapted to laboratory conditions for 24 h, then exposed to gold-doped nanoplastics at the test nominal concentration of 10^8^ particles mL^−1^. Zooplankton (7–11 individuals per 100 mL) specimens were exposed for 24 h, freshwater mussels (three individuals per 500 mL) were exposed for 4 h in filtered lake water with environmental microalgae added (5 × 10^5^ cells mL^−1^ for *Daphnia longispina-galeata,* 5 × 10^3^ cells mL^−1^ for *Eudiaptomus padanus,* and 3 × 10^4^ cells mL^−1^ for *Unio elongatulus*). Microalgae were also independently exposed to the same nanoparticles test nominal concentration to investigate their direct interaction with the particles.

After the exposure, samples of zooplankton were collected manually one by one with glass Pasteur pipettes, while their feces and pseudofeces were recovered through filtration using 100 µm filter mesh to separate them from free nanoplastics in the exposure medium (Figure S1).

Mussels were manually removed from freshwater, weighed, washed, dissected, freeze-dried, chopped to homogenize, and their feces and pseudofeces were manually collected and washed in UPW by centrifugation (2000 RCF, 5 min) (Figure S2). All samples were frozen at −20 °C until analysis.

### 2.4. Determination of Gold Level on Different Biomatrices by ICP-MS

Freshwater samples collected at the end of exposure were thawed and vortexed. Then, 1000 µL were transferred into 15 mL PP tubes, 400 µL of concentrated aqua regia (HNO_3_:HCl 1:3 *v*/*v*) were added and the samples were digested in a heating block for 60 min at 80 °C.

The zooplankton samples were transferred into 15 mL PP tubes. Then, 400 µL of concentrated aqua regia (HNO_3_:HCl 1:3 *v*/*v*) were added, and the samples were digested in a heating block for 80 min at 80 °C. After the acidic digestion, 1000 µL of H_2_O_2_ (30% *w*/*w*) were added to both the zooplankton and media digests. The heating continued for an additional 45 min at 80 °C. Once cooled down to room temperature, 50 µL of internal standard solution (Y and In at 0.1 mg L^−1^) were added to all digests, and the volume was brought to 5 mL with UPW prior to ICP-MS analysis.

The filters (100 µm mesh) were soaked in situ in 15 mL PP tubes by adding 5000 µL of 8% (*v*/*v*) aqua regia containing 1 µg L^−1^ Y and In as internal standard. The tubes were sonicated for 5 min, then placed in a heating block and heated for 150 min at 80 °C to digest the material captured by the filters. Filters were removed from the tubes prior to ICP-MS analysis.

For mussels, freeze-dried soft tissues were homogenized by grinding with an agate mortar and pestle. A quantity of 200 mg of the dry powder was placed in the digestion vessel and 500 µL of H_2_O_2_ (30% *w*/*w*) was added to initiate the degradation of organic matter. After 10 min, 4 mL of aqua regia (HNO_3_:HCl 1:3 *v*/*v*) were added, the vessel was capped and transferred to the Discover SP-D microwave digestion system (CEM Corp., Kamp Lintfort, Germany). The following parameters were used: 5 min ramp up to 240 °C, 30 min hold at 240 °C and 300 W maximal power, followed by a cooling time of roughly 7 min, down to 50 °C.

On the other hand, after centrifugation, the feces and pseudofeces were transferred into 15 mL PP tubes and concentrated aqua regia (HNO_3_:HCl 1:3 *v*/*v*) was added to each sample. Digestion was performed for 80 min at 80 °C using a heating block, with the addition of 500 µL of H_2_O_2_ (30% *w*/*w*) during the digestion process. Once at room temperature, UPW and internal standard solution (Y and In at 0.1 mg L^−1^) were added to all digests to reach final concentrations of 1 µg L^−1^ In and Y, and 8% *v*/*v* aqua regia, prior to ICP-MS analysis.

Total Au determination was performed with an Agilent 7700x ICP-MS, as described for the characterization of gold-doped nanoplastics with the High Matrix Introduction (HMI) mode enabled, to increase the signal/noise ratio and to handle saline samples. The instrumental set-up was the same as already described for the evaluation of ‘free’ ultrasmall AuNPs (Table S1).

### 2.5. Electron Microscopy

Scanning and transmission electron microscopes (SEM and TEM) were used to gain insight into the interaction between the organisms (zooplankton and mussels) feces and pseudofeces, microalgae cells, and nanoplastics.

For SEM analysis, silicon buffers were modified to create a hydrophobic and electrostatically positive surface, as described in literature [27,28]. The modified chip was placed at the bottom of the exposure beaker for the duration of the experiment (24 h). After exposure, the chip was incubated for 30 min in 1% formaldehyde (SEM) or in 2% Karnovsky (TEM), washed for 10 min in UPW, and stored at room temperature until further analysis. SEM images were obtained using the FEI-NovaNanolab 600I microscope, by detecting the secondary electrons at a 5 kV acceleration voltage with 0.40 nA of aperture.

TEM (JEOL JEM-2100, JEOL, Basiglio, Italy) in conjunction with EDX (Brüker, Milano, Italy) was used at 120 kV to confirm the presence of gold-doped nanoplastics in a freshwater environment around the microalgae cells and in the zooplankton and mussels’ feces and pseudofeces samples, after separation from the exposure medium (filtered water from Lake Maggiore, Varese, Italy). Aliquots of 3 µL were manually deposited on 200 mesh Formvar (Agar Scientific, Rotherham, UK) carbon-coated copper grids, dried overnight in a desiccator and analyzed.

## 3. Results

### 3.1. Characterisation of Gold-Doped Polymeric Nanoparticles

Gold-doped nanoplastics, synthesized in 2020 and later used in 2023 exposure studies, were evaluated for stability—specifically in terms of gold content and size distribution—prior to exposure to the selected freshwater organisms.

The filtrate (<20 nm) of the stock suspensions of Au-PE, Au-PP, and Au-PVC was digested and analyzed by ICP-MS to obtain information on the presence of “free” gold nanoparticles. The results are expressed as a percentage of “free” AuNPs (% wt), which corresponds to the ratio of the gold concentration found in the filtrate to that present in the initial suspension. The tests revealed the presence of “free” AuNPs within the Au-PP stock corresponding to 2.1 ± 0.1%, while “free” AuNPs were not detected in the Au-PE (<LoQ = 0.05%) and Au-PVC (<LoQ = 0.08%) suspensions.

Additionally, it was necessary to evaluate the stability of the gold-doped nanoplastics in UPW and in the exposure medium. The tests were performed using spICP-MS and DLS.

**Figure 1:**
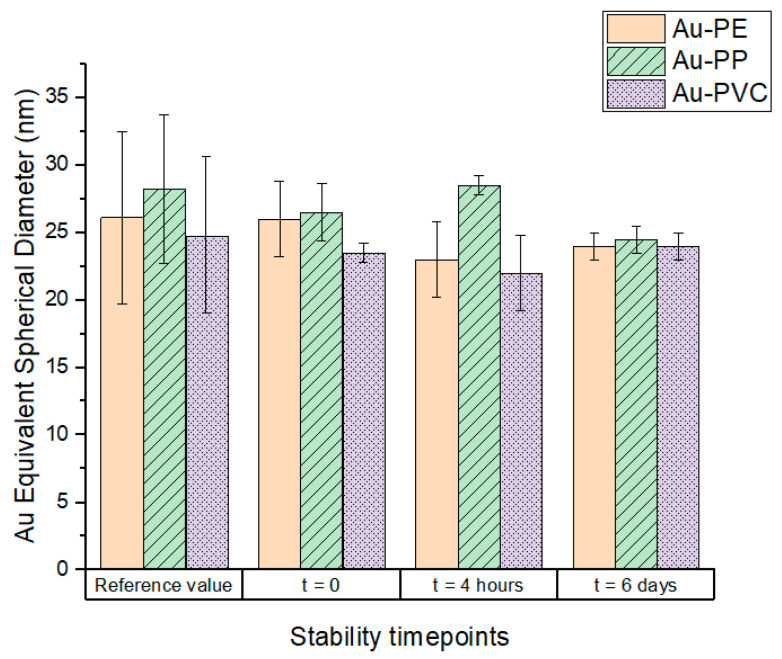
Au-based Equivalent Spherical Diameter (nm) of gold-doped nanoplastics synthesized at the JRC and stored for at least 41 months in terms of Au-based ESD before and after 4 h and 6 days in filtered lake water. Reference values from Cassano et al. 2021 and 2023 [23,24]. Values are presented as mean ± SD (*n* = 2 replicates).

The spICP-MS allowed the gold-equivalent spherical diameter (Au-based ESD) of gold-doped nanoplastics to be measured. Results presented in Figure 1 demonstrated that all three types of particles were stable in the stock solution after 41 months of storage (Figure 1, *t* = 0 in UPW) and in the lake water for at least 6 days. In particular, the size recovery, defined as the ratio between the measured Au-based ESD and the original characterization performed by Cassano et al. 2021 and 2023 [23,24], was very satisfactory, ranging from 94% to 100% for the three types of UPW-prepared particles. When the particles were dispersed in the exposure medium (filtered water from Lake Maggiore, Varese, Italy), the size recovery rates were also satisfactory, and ranged from 88% to 101% after 4 h, and from 87% to 97% after 6 days.

The spICP-MS stability test in freshwater showed that the particles remained fairly stable over 6 days in terms of gold equivalent spherical diameter, thus indicating little (or no) agglomeration/aggregation. However, since diameter is proportional to the cube root of mass, the dimer of a 26 nm particle (mass × 2) would theoretically be detected in spICP-MS as a 33 nm particle (ESD × 2^(1/3)^). An increase from 26 to 33 nm might be difficult to appreciate by spICP-MS, and therefore the presence of some dimers or trimers cannot be completely excluded.

**Figure 2:**
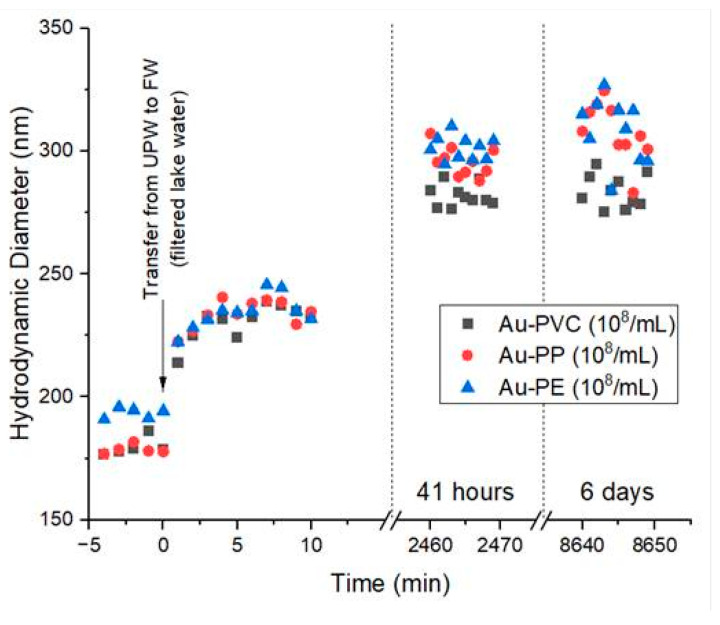
Assessment of aggregation or agglomeration of gold-doped nanoplastics in the exposure medium for 6 days.

To investigate potential aggregation or agglomeration of gold-doped nanoplastics in the exposure medium, DLS measurements were conducted over time (Figure 2). At *t* = 0, in UPW, the average particle sizes are 193 ± 2 nm for Au-PE, 179 ± 2 nm for Au-PP, and 180 ± 4 nm for Au-PVC. A rapid size increase (on average of hydrodynamic diameter, Dh, × 1.25 compared to *t* = 0) was observed within the first 10 min after transferring the particles from UPW to the filtered lake water. This initial increase likely resulted from particle coating with organic matter and/or some limited agglomeration/aggregation. After several days, the particle size increased further (on average Dh × 1.7 compared to *t* = 0) and remained relatively stable for up to 6 days. The increase in Dh of 170% would be compatible with the formation of dimers [29].

Finally, to correlate the nanoplastics number concentration with the mass of the gold, it was essential to determine the percentage of gold content and the mass of each particle.

The total gold content in each particle stock was quantified using ICP-MS and correlated with the solid content, calculated from the dry weight of the particle residue relative to the wet weight of the particle suspension. As a result, the mass percentages of gold associated with Au-PE, Au-PP, and Au-PVC particles were determined to be 5.1%, 4.1%, and 2.6%, respectively.

By considering the density of each type of polymer particle (Table S3), including the corresponding percentage of gold, the mass of each particle was estimated, thus providing an indication of the number of particles per nanogram of gold (Table 1).

### 3.2. Detection of Gold Level as Proxy of Nanoplastics

The level of gold was assessed in the biomatrices, specifically, zooplankton, mussels and their feces and pseudofeces, by ICP-MS.

**Figure 3:**
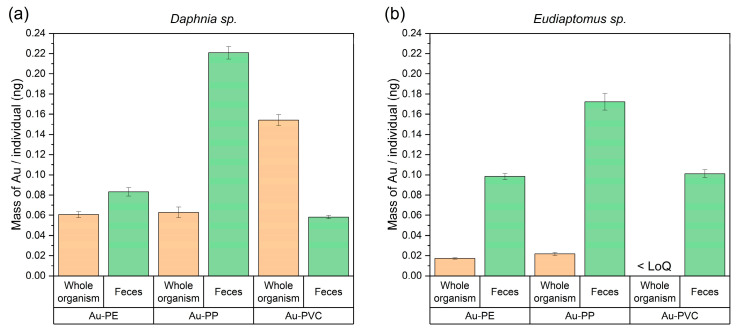
Mass of gold (as a proxy of Au-PE, Au-PP and Au-PVC particles) measured in feces and pseudofeces (collected on 100-µm mesh filters) and whole organisms relative to the number of individuals in the tanks for *D. longispina-galeata* (**a**) and *E. padanus* (**b**). The mean values and uncertainties presented are solely analytical (*n* = 3 replicate measurements of the same sample).

The data shown in Figure 3 revealed different gold concentrations accumulated by the two zooplankton species. In *D. longispina-galeata* group, the Au mass within the whole organism after one day of exposure ranged from 0.06 to 0.15 ng. In contrast, gold was detected at a lower level in *Eudiaptomus padanus* and only when exposed to Au-PE and Au-PP particles. Conversely, the Au contents in the feces and pseudofeces were similar in the *E. padanus* and *D. longispina-galeata group*, ranging from 0.06 to 0.22 ng.

When examining the relationship between the gold content introduced at an exposure dose of 10^8^ particles mL^−1^ and the levels detected within the organisms (uptake) and in their feces (excretion), both aquatic species were found to exhibit low gold concentrations. Specifically, gold levels ranged from 0.032% to 0.113% in *D. longispina-galeata* group and from 0.008% to 0.132% in *E. padanus* (Figure S3).

**Figure 4:**
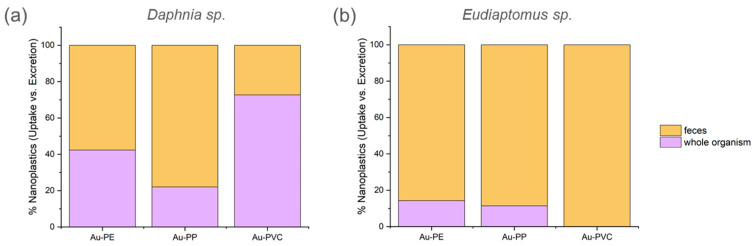
Percentage of gold-doped polymeric particles retained and excreted from *D. longispina-galeata* group (**a**) and *E. padanus* (**b**).

In *D. longispina-galeata group,* the percentage of gold absorbed (and therefore retained as particles in the tissues) was similar to the amount excreted in feces and pseudo-feces; in particular, for Au-PE and Au-PP particles. By contrast, *E. padanus* retained fewer particles and excreted a higher proportion of gold for all the polymer types (Figure 4).

**Figure 5:**
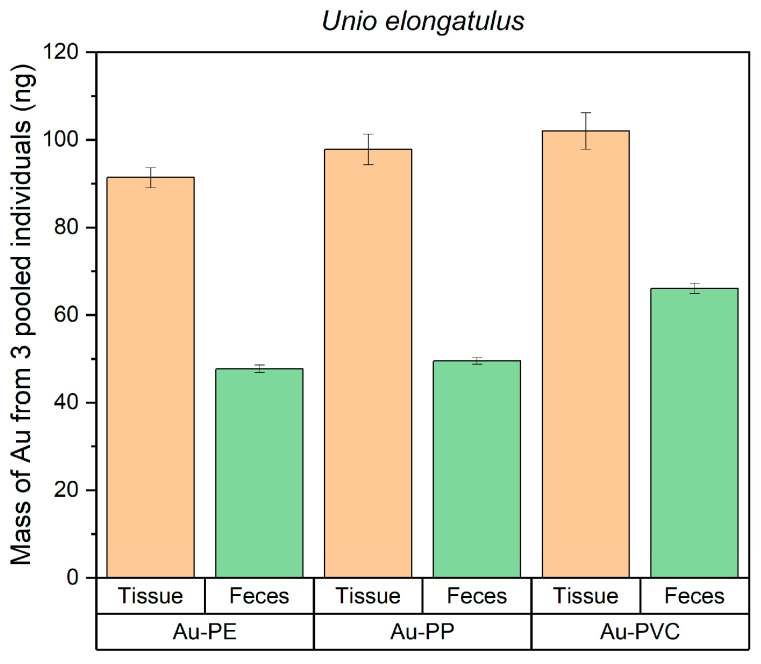
Mass of gold (as a proxy of Au-PE, Au-PP and Au-PVC particles) measured in mussels’ tissue and pseudofeces/feces after exposure.

After a 4 h exposure to gold-doped nanoplastics, the levels of gold detected in *U. elongatulus* soft tissues were higher than in the feces and pseudofeces for each type of polymeric gold-doped particle (Figure 5).

**Figure 6:**
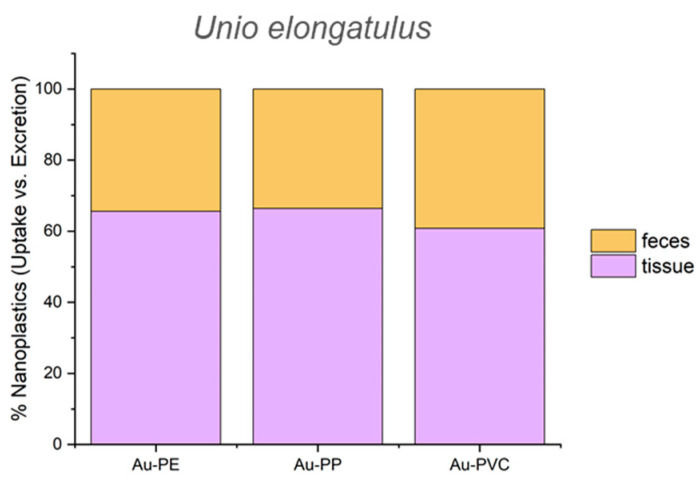
Percentage of gold-doped polymeric particles retained and excreted from *U. elongatulus*.

However, a slight increase in gold levels, and consequently in the number of particles, across the polymers (PE < PP < PVC) was observed in both the bivalves and their feces and pseudofeces. Between 2000 and 3000 billion particles were absorbed by the three individuals. Considering that 50,000 million particles were initially introduced into the exposure medium, the absorption rate for the three mussels could range from 4% to 6%. Using the same approach as described above for assessing the percentage levels of uptake versus excretion, higher levels of Au-NPLs were found for mussels than for zooplankton (Figure S3). Furthermore, the ratio of retained to ejected particles remained constant for all the three polymer types, with an approximate distribution of 64% ± 3% retained and 36% ± 3% ejected (Figure 6).

### 3.3. Examining Gold–Polymer Particle Interactions with Biomatrix via Electron Microscopy

**Figure 7:**
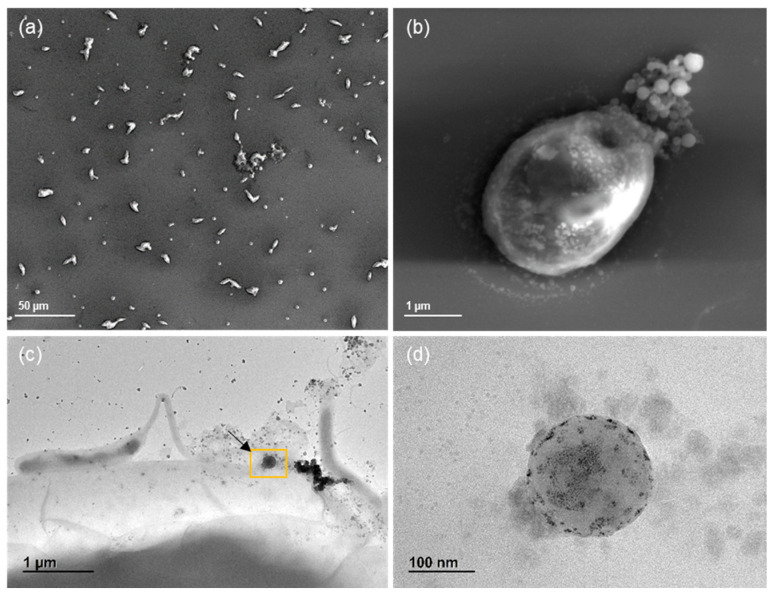
SEM (**a**,**b**) and TEM (**c**,**d**) micrographs at varying magnifications showing microalgae exposed to Au-PVC nanoplastics. Higher magnification (yellow square) in the TEM images confirms the presence of Au nanoparticles, visible as black dots.

The interaction between microalgae and the nanoparticles was examined using electron microscopy. Specifically, SEM images of the modified silicon chip captured a detailed “snapshot” of the exposure conditions. As illustrated in Figure 7a, microalgae exposed to nanoparticles for 24 h exhibited a partial colonization of their external surface. Following colonization, microalgae often develop a matrix of extracellular polymeric substances (EPS), which facilitates particle aggregation. This process may enable the co-transport of nanoparticles with microalgae, potentially enhancing their transfer to higher trophic levels. To confirm the presence of nanoplastics in the microalgae samples, TEM screening including EDX analysis was also performed (Figure 7).

**Figure 8:**
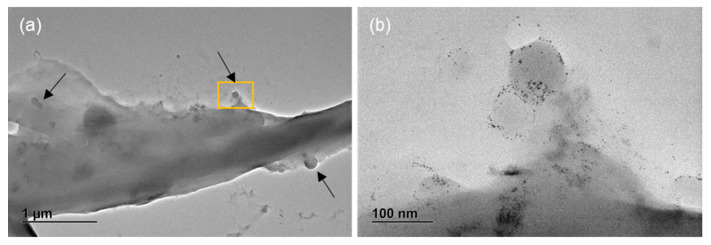
TEM micrographs of mussel feces and pseudofeces after exposure to Au-PVC nanoplastics. Arrows indicate particles trapped within the mussels’ feces and pseudofeces matrix. TEM images at lower (**a**) and higher (**b**) magnification confirm the presence of Au nanoparticles, visible as black dots.

Additional TEM analysis of mussel feces and pseudofeces revealed also significant interactions between gold-doped nanoplastics and the complex biological matrices. These interactions suggest that mussels, through their filter-feeding activity, can capture and incorporate nanoparticles into their feces and pseudofeces, potentially influencing the fate and transport of nanoplastics in aquatic ecosystems (Figure 8).

## 4. Discussion

Quantifying accumulation in biotic and abiotic compartments represents a major challenge in monitoring the distribution of nanoplastics in the ecosystem. In this study, metal-doped nanoplastics were used as model materials to track their behavior in complex exposure systems. Reliable analytical techniques, such as ICP-MS, were employed to monitor these particles and to formulate hypotheses regarding their potential uptake and release by targeted aquatic organisms. The stability of nanoplastics in aquatic environments is crucial for understanding their persistence and to forecast their potential impact on ecosystem integrity. Stability, bioaccumulation, and potential aggregation/agglomeration of gold-doped nanoplastics in freshwater organisms were investigated for the first time. Since the gold-doped nanoplastics used in this study were synthesized and stored for extended periods, their behavior in the stock suspension and exposure medium (filtered lake water) was closely monitored to avoid introducing an unaccountable source of bias in the study of nanoplastics partitioning. Gold-doped nanoplastics were characterized for their size distribution and gold content before and after exposure to various environmental conditions. All the three types of nanoplastics remained stable in UPW for up to 41 months. This suggests that the gold-doped nanoplastics were chemically stable over long storage periods, which is important when considering using them for future long-term experiments. When exposed to filtered lake water, the nanoplastics exhibited slight aggregation over time, which is consistent with the behavior of many nanoparticles in natural environments. The DLS measurements showed an initial rapid increase in particle size, followed by a more gradual increase over several days. This increase in size can be attributed to the interaction with dissolved species such as electrolytes and molecules. This can influence the surface charge of colloidal particles, increasing their possibility of aggregation [30,31]. The increase in size, up to 1.7 times the original size, suggests the formation of dimers and trimers; a common phenomenon observed when nanoparticles interact with organic materials in the environment [32].

In the context of nanoparticle exposure, a non-axenic microalgae culture was used as a food vector. A microalgae–nanoparticles interaction was observed through electron microscopy, suggesting that particle adsorbance on microalgae surface could play a key role in driving the fate and bioavailability of nanoparticles in the water column. Understanding how microalgae respond to nanoparticle exposure helps in predicting the potential for nanoparticles to enter the food chain and affect the productivity and integrity of freshwater systems.

Foodborne exposure of zooplankton and mussels to nanoparticles revealed significant differences in the uptake and distribution of nanoplastics between species. This variation in bioaccumulation likely results from differences in their feeding strategies. *D. longispina-galeata,* a non-selective filter-feeding animal within the nanoplankton size range (2–20 µm), accumulates higher levels of gold-doped nanoplastics (3.3 × 10^5^ of Au-PE, 5.3 × 10^5^ of Au-PP and 1.4 × 10^6^ of Au-PVC per individual) than *E. padanus* (9.3 × 10^4^ of Au-PE and 1.8 × 10^5^ of Au-PP per individual). On the other hand, copepods possess a greater ability to discriminate between different food items. These results align with previous studies that have demonstrated greater bioaccumulation of nanoplastics in cladocerans compared to copepods, due to the former’s more indiscriminate feeding behavior [33,34]. Additionally, the presence of gold in the feces and pseudofeces of both species suggests that elimination of the nanoplastics also occurs, albeit at a slower rate, indicating that the particles are not fully absorbed or metabolized within the organisms.

In freshwater mussels (*U. elongatulus*), the bioaccumulation of gold-doped nanoplastics was more pronounced than in zooplankton (4.9 × 10^8^ of Au-PE, 8.3 × 10^8^ of Au-PP and 9.0 × 10^8^ of Au-PVC per individual). These findings suggest that mussels are more likely to retain polymeric particles, as nanoplastics, rather than expel them. This aligns with their filter-feeding behavior, which enables them to process large volumes of water and trap particles, including nanoplastics, in their gills and tissue [35].

## 5. Conclusions

This study provides valuable insights into the stability, aggregation and bioaccumulation of gold-doped nanoplastics in freshwater organisms. While the particles remained stable over time and showed limited aggregation, certain freshwater species, particularly *D. longispina-galeata* and *U. elongatulus* efficiently accumulated these.

Within this framework, the combination of ICP-MS for quantitative analysis and electron microscopy for structural confirmation offers a comprehensive approach to studying nanoplastics uptake, distribution, and potential interactions in aquatic ecosystems.

SEM and TEM were crucial in confirming the interactions of the nanoplastics with microalgae and feces/pseudofeces. These techniques provided detailed images that revealed how nanoparticles can be transported to or from their surroundings as they are trapped in different biological matrices. This information helps to assess the processes by which nanoparticles are internalized, distributed, and potentially excreted or transferred to other parts of the body. Furthermore, ICP-MS analysis enabled precise quantification of nanoparticle concentrations in both the organisms and their surrounding environment, offering valuable insights into bioaccumulation. This evidence provides the scientific community with a better understanding of the levels of nanoparticles in aquatic organisms, shedding light on their potential impact in aquatic ecosystems.

The uptake of nanoparticles by these target organisms is of particular interest because they are prey for higher trophic levels, including fish; and therefore can serve as vectors for the transfer of nanoparticles within the food web. Further studies are needed to explore the long-term effects of nanoplastics exposure on aquatic organisms, and to assess the potential for trophic transfer in aquatic food webs.

## Figures and Tables

**Table 1:** Correlation between gold content and number of particles.

Particle Type	Particle Size ^1^ (nm)	Particle Mass ^2^ (ag)	Average Number of Particles per 1 ng of Au Detected ^3^
Au-PE	193 ± 2	3640	5.4 × 10^6^
Au-PP	179 ± 2	2874	8.5 × 10^6^
Au-PVC	180 ± 4	4382	8.8 × 10^6^

^1^ Measured by DLS in UPW and expressed as hydrodynamic diameter (Dh) Z-average. ^2^ Calculated taking into account density of gold-doped polymeric particles with specific % wt of Au; attogram (ag). ^3^ Estimate taking into account the % wt of Au per particle and the mass of each particle.

## Data Availability

Data is unavailable due to privacy or ethical restrictions.

## Supplementary Materials

The following supporting information can be downloaded at: https://www.mdpi.com/article/10.3390/nano15020116/s1, Figure S1: Experimental setup for zooplankton exposure to gold-doped nanoplastics (Au-PVC, Au-PE and Au-PP) and corresponding sample collection; Figure S2: Experimental setup for mussel exposure to gold-doped nanoplastics (Au-PVC, Au-PE and Au-PP) and corresponding sample collection. Figure S3: Mass percentage of particles within freshwater zooplankton (A and B) and mussels (C) and in their feces/pseudofeces, relative to the initial gold reference value for the exposure dose (10^8^ particles mL^−1^); Table S1: Agilent 7700x detailed instrument and method configuration for gold (Au) analysis; Table S2: Perkin Elmer Nexion 300D detailed instrument and method configuration for gold analysis in spICP-MS mode; Table S3: Selected characteristics of gold-doped nanoplastics (Au-PVC, Au-PE and Au-PP) and ultrasmall gold nanoparticles (Au-NPs).
