# Supplementary material for: Exploring Nanoplastics Bioaccumulation in Freshwater Organisms: A Study Using Gold-Doped Polymeric Nanoparticles
Source: Nanomaterials (Basel). 2025 Jan 15;15(2):116. doi: 10.3390/nano15020116 (PMC11767279; doi:10.3390/nano15020116)
Supplement: Supplementary file 1 [file nanomaterials-15-00116-s001.zip › nanomaterials-3404674-supplementary.pdf]

Supplementary Information

Article

Exploring Nanoplastics Bioaccumulation in Freshwater Organisms: A Study Using Gold-Doped Polymeric Nanoparticles

Gabriella F. Schirinzi [1], Guillaume Bucher [1], Marisa Sárria Pereira de Passos [1,‡], Vanessa Modesto [2], Miguel-Ángel Serra [1], Douglas Gilliland [1], Nicoletta Riccardi [2] and Jessica Ponti [1,*]

[1] European Commission, Joint Research Centre (JRC), Ispra, Italy; gabriella.schirinzi@ec.europa.eu (G.F.S.); guillaume.bucher@ec.europa.eu (G.B.); m.sarria@biotec.rwth-aachen.de(M.S.P.d.P.); miguel.serra-beltran@ec.europa.eu (M.A.S.); douglas.gilliland@ec.europa.eu (D.G.)

[2] Water Research Institute (IRSA), National Research Council (CNR), 28922 Pallanza, Italy; vane.modesto@gmail.com (V.M.); nicoletta.riccardi@irsa.cnr.it (N.R.)

‡ current affiliation: m.sarria@biotec.rwth-aachen.de (M.S.P.d.P.)

* Correspondence: jessica.ponti@ec.europa.eu

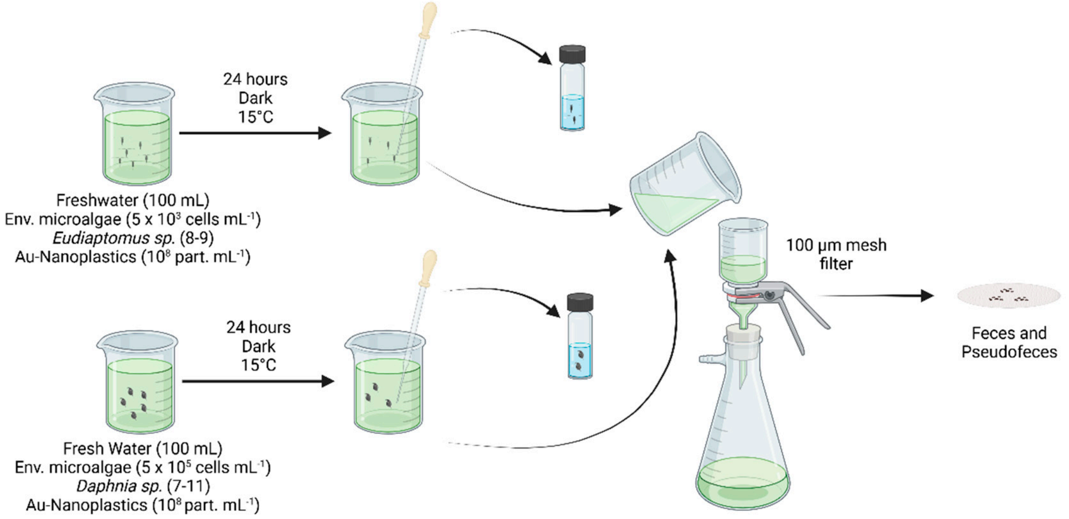

Figure S1. Experimental setup for zooplankton exposure to gold-doped nanoplastics (Au-PVC, Au-PE, and Au-PP) and corresponding sample collection.

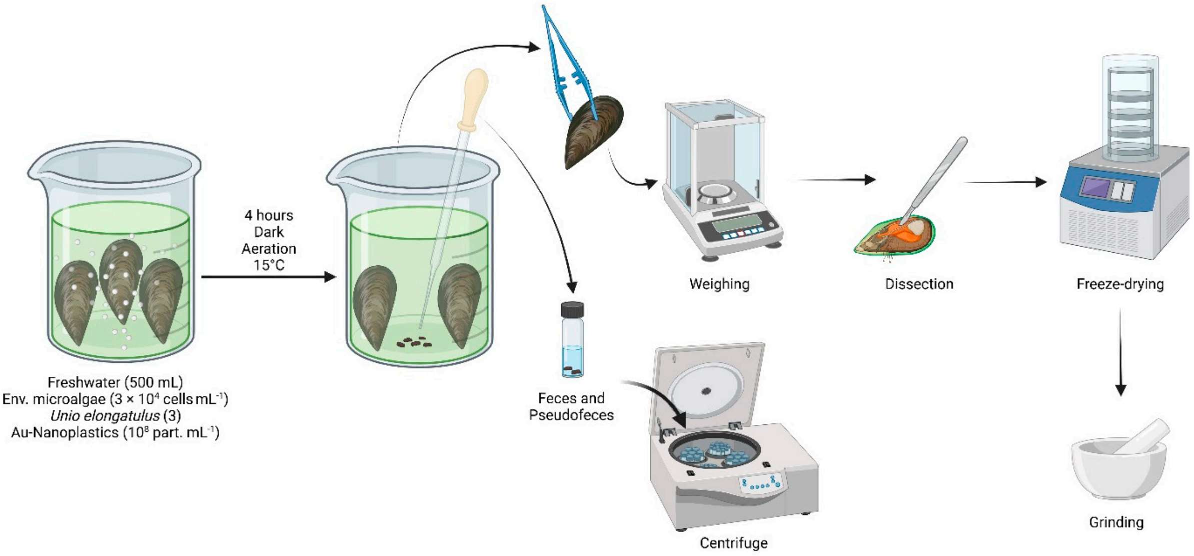

Figure S2. Experimental setup for mussel exposure to gold-doped nanoplastics (Au-PVC, Au-PE, and Au-PP) and corresponding sample collection.

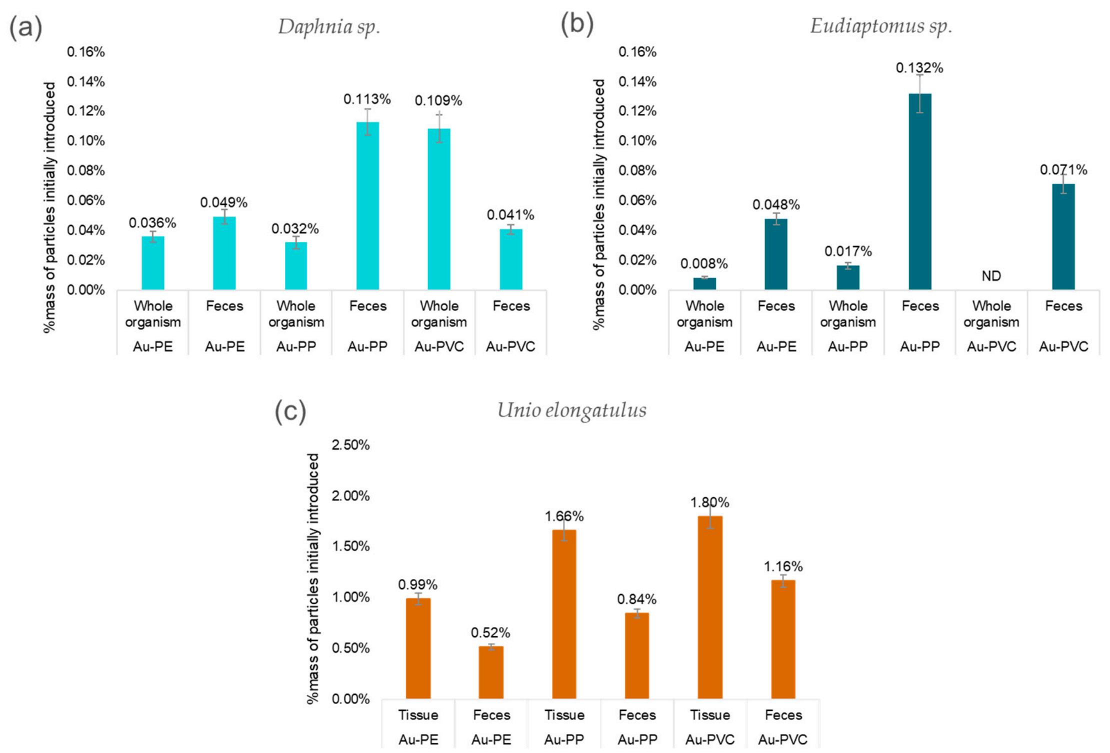

Figure S3. Mass percentage of particles within freshwater zooplankton (**a** and **b**) and mussels (**c**) and in their feces and pseudofeces, relative to the initial gold reference value for the exposure dose (10^8 particles mL^{-1}).

Table S1: Agilent 7700x detailed instrument and method configuration for gold (Au) analysis.

Parameter	Value
**Introduction system**	Micromist nebulizer 0.4 mL min-1
	Scott double-pass spray chamber set at 2 °C
	Quartz torch with 2.5 mm id injector
	Platinum sampling and skimmer cones
	Uptake tubing Tygon 1.02 mm id (white/white)
	Drain tubing PharMed 1.52 mm id (yellow/blue)
**Plasma power**	1550 W
**Sampling depth**	8 mm
**Plasma gas flow (Ar)**	15 L min^{-1}
**Auxiliary gas flow (Ar)**	0.9 L min^{-1}
**Nebulization (carrier) gas flow (Ar)**	0.7 L min^{-1}
**HMI (dilution) gas flow (Ar)**	0.4 L min^{-1}
**Target Analyte**	^{197}Au
**Internal Standard (ISTD)**	^{115}In (as primary internal standard)
**Other elements monitored**	^{89}Y (as secondary internal standard)
**Wash between injections (Rinse 1)**	5 % HNO_3
**Probe rinse port**	H_2O

Table S2: Perkin Elmer Nexion 300D detailed instrument and method configuration for gold analysis in spICP-MS mode.

Parameter	Value
**Introduction system**	Meinhard concentric nebulizer
	Glass cyclonic spray chamber set at RT
	Quartz torch with 2.5 mm id injector
	Nickel sampling and skimmer cones
	Uptake tubing PVC 0.38 mm id
	Drain tubing Santoprene 1.30 mm id
**Plasma power**	1600 W
**Plasma gas flow (Ar)**	18 L min^{-1}
**Auxiliary gas flow (Ar)**	1.2 L min-1
**Nebulization (carrier) gas flow (Ar)**	1.0[a] L min^{-1}
**Sample uptake**	0.167[b] mL min^{-1}
**Transport Efficiency**	6.10 %c
**Target Analyte**	^{197}Au
**Wash between injections**	2 % HNO_3 then H_2O

[a]Optimized daily
[b]Measured gravimetrically daily
[c]Determined daily

Table S3: Selected characteristics of gold-doped nanoplastics (Au-PVC, Au-PE and Au-PP) and ultrasmall gold nanoparticles (Au-NPs).

Particles	Plastic Content[1] (% wt)	Solid Content[2] (% wt)	[Au][3] (mg L^{-1})	Au for particles (% wt)[4]	Density (g cm^{-3})[5]	Au-based ESD[6] (nm)
Au-PVC	0.283 ± 0.001	0.23 ± 0.01	60.6 ± 0.7	2.6	1.435	24.8 ± 5.8
Au-PE	0.178 ± 0.002	0.20 ± 0.01	101.0 ± 1.3	5.1	0.962	26.1 ± 6.4
Au-PP	0.224 ± 0.002	0.26 ± 0.01	105.6 ± 0.3	4.1	0.967	28.2 ± 5.5
Au-NPs	N/A	N/A	10807 ± 97			N/A

[1]Measured by TOC – All organic carbon assumed to come from plastic
[2]Measured as = dry weight of particle residue / wet weight of particle suspension
[3]Measured by ICP-MS after acidic digestion
[4]Calculated taking into account dry weight of particle residue and Au content measured by ICP-MS
[5]Estimated as $\rho_{AuNPLs} = \rho_{Au} \cdot V_{Au} + \rho_{polymer} \cdot V_{polymer.}$ Where ρ is the density and V is the volume of gold and polymeric particles that compose the gold-polymeric nanoparticles (Au-NPLs)
[6]Equivalent Spherical Diameter (ESD) measured by spICP-MS (Cassano *et al.* 2021 and 2023)
